# TNF-α Mediates Diabetes-Enhanced Chondrocyte Apoptosis During Fracture Healing and Stimulates Chondrocyte Apoptosis Through FOXO1

**DOI:** 10.1002/jbmr.59

**Published:** 2010-02-08

**Authors:** Rayyan A Kayal, Michelle Siqueira, Jazia Alblowi, Jody McLean, Nanarao Krothapalli, Dan Faibish, Thomas A Einhorn, Louis C Gerstenfeld, Dana T Graves

**Affiliations:** 1 Department of Periodontology and Oral Biology, Boston University School of Dental MedicineBoston, MA, USA; 2 Department of Orthopedic Surgery, Boston University School of MedicineBoston, MA, USA; 3 Department of Periodontics, University of Medicine and Dentistry of New JerseyNewark, NJ, USA

**Keywords:** apoptosis, bone, cell death, chondrocyte, cartilage, cytokine, fracture, forkhead, nuclear localization, transcription factor

## Abstract

To gain insight into the effect of diabetes on fracture healing, experiments were carried out focusing on chondrocyte apoptosis during the transition from cartilage to bone. Type 1 diabetes was induced in mice by multiple low-dose streptozotocin injections, and simple transverse fractures of the tibia or femur was carried out. Large-scale transcriptional profiling and gene set enrichment analysis were performed to examine apoptotic pathways on total RNA isolated from fracture calluses on days 12, 16, and 22, a period of endochondral bone formation when cartilage is resorbed and chondrocyte numbers decrease. Tumor necrosis factor α (TNF-α) protein levels were assessed by ELISA and caspase-3 by bioactivity assay. The role of TNF was examined by treating mice with the TNF-specific inhibitor pegsunercept. In vitro studies investigated the proapoptotic transcription factor FOXO1 in regulating TNF-induced apoptosis of chondrogenic ATDC5 and C3H10T1/2 cells as representative of differentiated chondrocytes, which are important during endochondral ossification. mRNA profiling revealed an upregulation of gene sets related to apoptosis in the diabetic group on day 16 when cartilage resorption is active but not day 12 or day 22. This coincided with elevated TNF-α protein levels, chondrocyte apoptosis, enhanced caspase-3 activity, and increased FOXO1 nuclear translocation (*p* < .05). Inhibition of TNF significantly reduced these parameters in the diabetic mice but not in normoglycemic control mice (*p* < .05). Silencing FOXO1 using siRNA in vitro significantly reduced TNF-induced apoptosis and caspase activity in differentiated chondrocytes. The mRNA levels of the proapoptotic genes *caspase-3*, *caspase-8*, *caspase-9*, and *TRAIL* were significantly reduced with silencing of FOXO1 in chondrocytic cells. Inhibiting *caspase-8* and *caspase-9* significantly reduced TNF-induced apoptosis in chondrogenic cells. These results suggest that diabetes causes an upregulation of proapoptotic genes during the transition from cartilage to bone in fracture healing. Diabetes increased chondrocyte apoptosis through a mechanism that involved enhanced production of TNF-α, which stimulates chondrocyte apoptosis and upregulates mRNA levels of apoptotic genes through FOXO1 activation. © 2010 American Society for Bone and Mineral Research.

## Introduction

Diabetes has as one of its complications osteopenia associated with decreased bone mineral density (BMD).(1–6) Several mechanisms have been suggested for osteopenia caused by type 1 diabetes, including reduced bone formation supported by deceased levels of serum osteocalcin.(6) It also may be due to increased bone resorption, indicated by enhanced serum levels of type 1 collagen cross-linked carboxy-terminal telopeptide.(1) Alternatively, reduced numbers of osteoblasts and decreased production of extracellular matrix proteins associated with decreased production of growth factors such as insulin-like growth factor 1 (IGF-1) may account for diminished bone formation.(1,2) Animal models are consistent with human studies demonstrating increased basal levels of bone resorption associated with increased expression of TRACP and cathepsin K, as well as decreased markers of bone formation such as alkaline phosphatase.(7,8)

Diabetes has been shown to significantly impair fracture healing. Case reports and clinical investigations have reported delayed union or increased healing time in diabetic subjects compared with matched controls.(9,10) It has been reported that diabetic animals had a significantly smaller amount of new bone on days 14 and 21 after fracture than normal and insulin-treated animals, which was associated with decreased Ca deposition.(11) The DNA content of diabetic fracture calluses is decreased by 40% compared with normal controls.(9) This is an indication that the diabetic calluses have decreased cellularity compared with normal calluses. In addition, the same study reported reduced bone matrix formation, represented by a 50% to 55% decrease in the collagen content of the calluses of diabetic animals. Diabetic animals have significantly smaller calluses with smaller bone and cartilage areas than normoglycemic animals.(12) In addition to having an impact on bone formation, diabetes affects the transition from cartilage to bone during fracture repair by accelerating the loss of cartilage, which serves as an anlage for endochondral bone formation.(13,14) The greater loss of cartilage in diabetic fracture calluses is associated with increased mRNA levels of proresorptive factors and diabetes-enhanced osteoclastogenesis.(13)

Chondrocyte apoptosis is an important event in the transition from cartilage to bone during fracture healing and growth of long bones.(15) This process is induced by cytokines such tumor necrosis factor α (TNF-α) and interleukin 1β (IL-1β).(16,42) Chondrocytes have been shown to produce RANKL and may regulate osteoclastogenesis at growth plates to remove calcified matrix in response to bone morphogenetic protein 2 (BMP-2).(17) Apoptosis of chondrocytes may be important for matrix degradation.(16) Apoptosis and the associated activation of the caspase proteolytic cascade has been detected in parallel with matrix metalloproteinase (MMP) expression and may be one mechanism for the activation of MMPs.(17) Moreover, apoptosis of hypertrophic chondrocytes is associated with secretion of osteogenic growth factors such as transforming growth factor β (TGF-β) and angiogenic factors such as fibroblast growth factor 2 (FGF-2) and may be important in regulating the early steps in bone formation as cartilage matrix is resorbed.(18) Similarly, apoptotic vesicles produced by chondrocytes are rich in alkaline phosphatase and may regulate the calcification of osteoid matrix.(17)

Diabetes enhances the expression of proinflammatory factors in a number of conditions. In some cases, this is tied to increased NF-κB activity, which is directly antiapoptotic. In contrast, the transcription factor FOXO1 is proapoptotic and stimulated under conditions present in diabetes, such as increased levels of inflammatory mediators, advanced glycation end products, and reactive oxygen species and decreased Akt activity.(19–21) Our laboratory has shown that diabetes increases FOXO1 DNA binding activity during fracture healing and that diabetes enhances FOXO1 nuclear translocation in chondrocytes in vivo.(22) In numerous cell types, activation of the FOXO family leads to apoptosis, particularly when its expression or activation is prolonged.(23,24) Some of the FOXO-responsive genes are apoptotic effectors such as TNF-α and FAS ligand.(19,25)

To gain insight into the effect of diabetes on fracture healing, large-scale transcriptional profiling analysis and gene set enrichment analysis were performed on total mRNA isolated from fracture calluses at various stages during fracture healing in control and diabetic mice. The effect of diabetes on the expression of various functional ontologies of gene expression then was assessed by gene set enrichment analysis (GSEA) focusing on apoptotic pathways. In order to validate changes in apoptotic mechanisms in chondrocyte, tissues were examined by histologic assessment. The potential regulatory role that TNF-α carries in mediating accelerated chondrocyte apoptosis was investigated by treating mice with the TNF-specific inhibitor pegsunercept. Finally, since TNF-induced apoptosis is mediated through FOXO1 in fibroblasts and osteoblasts and diabetes increases FOXO1 activity,(19,22,26) we examined the relationship between FOXO1 and chondrocyte apoptosis in vitro using siRNA.

## Materials and Methods

### Induction of type 1 diabetes

All experiments were approved by the Boston University Medical Center Institutional Animal Care and Use Committee (IACUC). Eight-week-old male CD-1 mice purchased from Charles River Laboratories (Wilmington, MA, USA) were rendered diabetic by an intraperitoneal (i.p.) injection of streptozotocin (40 mg/kg; Sigma, St. Louis, MO, USA) in 10 mM of citrate buffer daily for 5 days.(27) Control mice were treated identically with vehicle alone, 10 mM of citrate buffer. A group of diabetic mice was treated with the TNF-α inhibitor pegsunercept by i.p. injection (4 mg/kg) every 3 days starting on day10 after fracture until the time of euthanasia. Animals were considered to be diabetic when serum glucose levels exceeded 250 mg/dL (Accu-Chek, Roche Diagnostics, Indianapolis, IN, USA). Glycosylated hemoglobin levels were measured at the time of euthanasia by Glyco-tek Affinity Chromatography (Helena Laboratories, Beaumont, TX, USA). The diabetic group had values that were typically approximately 12%, and normoglycemic mice had values that were approximately 6%. Average blood glucose levels of diabetic animals were approximately three times higher than the values found in normoglycemic mice.

### Tibial and femoral fractures

All studies were performed on male mice that were diabetic for at least 3 weeks prior to fracture. A simple transverse closed fracture of the tibia or femur was performed in separate animals, as described previously.(13,28,29) For tibial fractures, an incision was made on the medial aspect of the knee, and the articular surface of the tibia was exposed. In the femoral fractures, the incision was made lateral to the knee, and the tendon for the quadriceps femoris muscle was pushed medially, exposing the articular surface of the femur. Access to the medullary canal was gained with a 25-gauge needle, and a 27-gauge spinal needle was inserted for fixation. After closure of the incision, a fracture was created by blunt trauma. Fractures were examined radiographically, and any fractures not consistent with standardized placement criteria (mid-diaphyseal) or grossly comminuted were excluded. Animals were subsequently euthanized at 12, 16, and 22 days after fracture for the tibias and after 10, 16, and 22 days after fracture for the femurs.

### Histology and histomorphometric analysis

The femurs with a small amount of surrounding muscle and soft tissues were fixed for 72 hours in cold 4% paraformaldehyde and decalcified for 2 weeks by incubation in cold Immunocal (Decal Corporation, Congers, NY, USA). After decalcification, specimens were embedded in paraffin, sectioned at 5 µm, and prepared for staining. Apoptotic cells were detected by the TUNEL assay (ApopTag Peroxidase In Situ Apoptosis Detection Kit, Chemicon International, Temecula, CA, USA) on sections stained with safranin-O/fast green to distinguish cartilage. For each data point, there were six to eight specimens. One examiner under blinded conditions made the measurements, with the results confirmed by a second examiner.

### FOXO1 nuclear localization

Sections were prepared as described earlier and incubated overnight with anti-FOXO1 antibody (Santa Cruz Biotech, Santa Cruz, CA, USA) or matched negative control antibody. Primary antibody to FOXO1 was detected by a Cy5-tagged secondary antibody. Propidium iodide nuclear stain was included in the mounting medium. FOXO1 nuclear localization was detected by confocal laser scanning microscopy at a focal plane that bisected the nuclei (Axiovert-100M, Carl Zeiss, Thornwood, NY, USA). Cy5, propidium iodide, and phase-contrast images (original magnification ×400) of the same field were captured digitally. The cartilage area in nonoverlapping fields was analyzed, and the percent chondrocytes with unambiguous nuclear localization was assessed by comparing FOXO1/Cy5, propidium iodide, and merged and phase-contrast images. For each group, *n* = 5 to 6 specimens. Results were confirmed by a second examiner.

### mRNA profiling of gene sets that regulate apoptosis

After euthanasia, fractured tibial calluses were carefully dissected, removing all muscle and noncallus tissue, and immediately frozen in liquid nitrogen. Total RNA was extracted with Trizol (Life Technologies, Rockville, MD, USA) from pulverized frozen tissue and further purified by an RNAeasy MinElute Cleanup Kit (Qiagen, Valencia, CA, USA). The concentration and integrity of the extracted RNA were verified by 260/280-nm spectrophotometry and denaturing agarose gel electrophoresis with ethidium bromide staining.

mRNA profiling was carried out using a PGA Mouse Version 1.1 array (MGH-ParaBioSys, Boston, MA, USA). Microarray probe preparation, hybridization, and reading of fluorescent intensity were performed by the Massachusetts General Hospital Microarray Core Facility (Cambridge, MA, USA). All slides were coprinted with an internal “alien” sequence that has no sequence homologues in the mouse genome. Slide printing, array labeling, hybridization, and slide reading were performed at the Massachusetts General Hospital Genomics Core Facility, as described previously.(30) All slides were quality control tested and contained appropriate positive and negative control sequences for data analysis. The alien gene in these studies serves as both a genome-extrinsic sequence and a universal in-spot reference. In the experiments reported here, all microarrays were printed in such a way that an alien 70mer probe was coprinted with each gene specific probe such that the alien was at a final concentration of 10% of the murine gene oligonucleotide. The data were normalized by scaling all individual intensities such that mean total intensities were the same for all comparative samples (normal, diabetic, and alien) within a single array and across replicates. Using the intensity reading, background was calculated locally per spot and subtracted from the intensity measurement of each hybridized spot. The ratio of normal to alien was calculated first. Using this ratio, all outliers for a given gene were discarded. The standard log_10_ of diabetic versus normal and diabetic versus alien for each spot was calculated, and the distribution of the log ratios was obtained from combined replicates per time point. The data are combined using the geometric mean of four replicate ratios.

Microarray data were analyzed using GSEA software and the Molecular Signature Database (MSigDB) as described in ref. (31), which can be accessed at the following Web site: http://www.broad.mit.edu/gsea/. Genes were first ranked based on the correlation between their expression and the class distinction. An enrichment score then was calculated that reflects the degree to which a gene is overrepresented at the extremes (top or bottom) of the entire ranked list. Statistical significance (*p* value) and false discovery rate (FDR) of that enrichment score then were calculated. This statistical analysis determines whether a gene set is significantly upregulated or downregulated in the experimental sample compared with a control. Gene sets with an FDR of less than 25% and a nominal *p* value of less than .05 were considered significant.(31) Genes that contributed to a significant difference in pathways related to apoptosis were identified by the GSEA software and further screened for significance by Student's *t* test (*p* <. 05), as well as for a threshold change of 1.5 increase or decrease in the diabetic versus normoglycemic microarray values, as described in ref. (32).

For *TNF-α*, *TRAIL*, and *caspase-4* mRNA levels obtained with microarrays were validated by real-time PCR using primers and probe sets purchased from Applied Biosystems (Foster City, CA, USA), as described previously.(19) Experiments were carried out on three sets of samples, and each set represented three animals for *n* = 9. For a given experiment, RNA was combined and TaqMan reagents were used for first-strand cDNA synthesis and amplification. Results were normalized with an 18S ribosomal primer and probe set. Each experiment was performed three times, and the results from the three separate experiments were combined to derive mean values. For a given gene, the expression for each group was set relative to the value obtained for the normoglycemic control animals on day 12.

### TNF-α and caspase-3 protein levels

Frozen tissue was pulverized and incubated with cold lysis buffers containing protease inhibitors (Pierce, Rockford, IL, USA) and tested for TNF-α (R&D, Minneapolis, MN, USA) and caspase-3 activity with a luminescent kit (Promega, San Luis Obispo, CA, USA). Each assay was carried out three times with similar results.

### In vitro experiments

In vitro experiments were carried out with cell lines that had a chondrogenic phenotype induced by BMP stimulation. Experiments were performed with ATDC5 (murine chondrogenic cell line) and C3H10T1/2 (murine mesenchymal stem cell line) cells obtained from the American Type Culture Collection (Rockville, MD, USA). Pluripotent C3H10T1/2 cells have been shown to undergo chondrogenic differentiation in the presence of BMP-2.(33,34) ATDC5 cells undergo multistep chondrogenic differentiation, in which they differentiate into type II and X collagen–expressing cells.(35,36) Thus, with BMP-2 stimulation, both C3H10T1/2 and ATDC5 cells exhibit a hypertrophic chondrocyte phenotype.(37) Cells were grown in a 1:1 mixture of DMEMand Ham's F-12 medium (Cellgro, Manassas, VA, USA) at 37°C in a humidified atmosphere of 5% CO_2_ in air. The medium was supplemented with 5% fetal bovine serum (FBS) for ATDC5 and 10% FBS for C3H10T1/2 and 1% penicillin/streptomycin. To induce differentiation into chondrocytes, ATDC5 cells were incubated in 0.5% FBS medium with 200 ng/mL BMP-2 (Peprotech, Rocky Hill, NJ, USA) for 6 days, whereas C3H10T1/2 cells were incubated in 0.5% FBS and 100 ng/mL BMP-2 for 4 days. The chondrogenic phenotype was verified by BMP-2-induced *collagen II* and *collagen* *X* mRNA levels measured by real-time PCR.

To inhibit reactive oxygen species (ROS), cells were plated in six-well plates and were preincubated with 5 or 10 mM of *N*-acetyl-l-cysteine (NAC) (Sigma-Aldrich) for 2 hours or with 5 or 10 mM of Trolox (vitamin E analogue; Calbiochem, Gibbstown, NJ, USA) for 30 minutes, followed by TNF-α stimulation (20 ng/mL) as described earlier. To inhibit caspases, cells were plated in six-well plates and were incubated with either 50 µM of caspase-8 inhibitor IETD-FMK (RandD Systems, Minneapolis, MN, USA), 50 µM of caspase-9 inhibitor LEHD-FMK (RandD Systems), or a mixture of both for 24 hours combined with 20 ng/mL of TNF-α.

ATDC5 and C3H10T1/2 cells were transferred to six-well plates and, when 70% confluent, were transfected with 148.5 ng/mL of FOXO1 siRNA or scrambled siRNA with HiPerFect (Qiagen, Valencia, CA, USA) in medium supplemented with 0.25% FBS for 24 hours. The target sequence of *FOXO* siRNA was CCA GCT ATA AAT GGA CAT TTA. After another 24 hours in low-serum medium, cells were stimulated with 20 ng/mL of TNF (Peprotech) for 6 hours. Total RNA was extracted using QIA shredder Mini Spin Columns and RNAeasy Mini Kit (Qiagen). MultiScribe Reverse Transcriptase (Applied Biosystems) was used to convert RNA to cDNA, and real-time PCR was performed using Taqman primers and probe sets (Applied Biosystems). Nuclear and cytoplasmic proteins were extracted from cell lysate using NE-PER Nuclear and Cytoplasmic Extraction Reagents Kit (Pierce), and a protease inhibitor cocktail was added (Pierce). Bicinchoninic acid protein assay (BCA) was used to measure protein content of the nuclear and cytoplasmic extract (Pierce). *FOXO1* DNA-binding activity was measured using a transcription factor ELISA kit for *FOXO1* (Active Motif, Carlsbad, CA) following the manufacturer's instructions. Apoptosis was measured by the use of a Cell Death Detection ELISA kit (Roche), which detects the amount of histone-associated DNA fragments in mono- and oligonucleosomes, which are known markers of apoptotic cells. Caspase-3/7 activity was measured by a luminometric substrate (Promega) following manufacturer's instructions.

### Statistical analysis

Data represent mean values ± SEM. Statistical significance between diabetic and normoglycemic groups for a given parameter was established by Student's *t* test at the *p* < .05 level. ANOVA with Scheffe's post hoc test was used to analyze differences between multiple groups at the same time point.

### Results

To assess the impact of diabetes on apoptosis in healing fractures, large-scale transcriptional profiling analysis and GSEA were performed on RNA isolated from fracture calluses at various stages during fracture healing in control and diabetic mice. A striking result was that on day 16 after fracture, diabetes enhanced 13 of 21 gene sets associated with apoptosis (Table 1). These gene sets were frequently associated with the extrinsic pathway, such as TNF and Fas. Gene sets associated with the intrinsic pathway tended to not be upregulated by diabetes, including the ceramide, p53, and BAD pathways. In contrast, on days 12 and 22 after fracture, diabetes had relatively little effect on upregulation of the apoptotic gene sets, suggesting that the major effects of altered apoptotic regulation occurred in the later hypertrophic period of chondrocyte differentiation, during endochondral bone formation that is seen in the callus tissues. Individual proapoptotic genes that significantly contributed to the entire set being upregulated in the diabetic group were identified by leading-edge analysis. Proapoptotic genes that appeared in more than three gene sets included *NFκBIα*, *TRAF-1*, *TRAF-2*, *TRAF-3*, *TNF-α*, *MCL-1*, *TNF receptor 1α*, *APAF-1*, and *caspase-1*.

**Table 1:** Gene Set Enrichment Analysis for Pathways Involved in Apoptosis

Normal vs diabetic		Day 12				Day 16				Day 22			
				
		Upregulated in normal		Upregulated in diabetic		Upregulated in normal		Upregulated in diabetic		Upregulated in normal		Upregulated in diabetic	
							
Apoptotic gene sets	No. of genes	*p* Value	FDR	*p* Value	FDR	*p* Value	FDR	*p* Value	FDR	*p* Value	FDR	*p* Value	FDR
Passerini apoptosis	34	N/S	N/S	N/S	N/S	N/S	N/S	0*	.07*	N/S	N/S	N/S	N/S
Apoptosis Kegg	37	N/S	N/S	N/S	N/S	N/S	N/S	0*	.11*	N/S	N/S	N/S	N/S
Apoptosis	57	N/S	N/S	N/S	N/S	N/S	N/S	0*	.13*	N/S	N/S	N/S	N/S
Death pathway	28	N/S	N/S	N/S	N/S	N/S	N/S	.01*	.19*	N/S	N/S	N/S	N/S
Cell death	13	N/S	N/S	N/S	N/S	N/S	N/S	N/S	N/S	N/S	N/S	N/S	N/S
SA programmed cell death	11	N/S	N/S	N/S	N/S	N/S	N/S	N/S	N/S	N/S	N/S	N/S	N/S
Vanasse BCL2 targets	77	0*	.01*	N/S	N/S	N/S	N/S	0*	.02*	0*	.21*	N/S	N/S
NF-κB pathway	21	N/S	N/S	N/S	N/S	N/S	N/S	0*	.09*	N/S	N/S	N/S	N/S
TNF and FAS network	19	N/S	N/S	N/S	N/S	N/S	N/S	0*	.10*	N/S	N/S	N/S	N/S
TNFA NF-κB dep up	16	N/S	N/S	N/S	N/S	N/S	N/S	0*	.07*	N/S	N/S	N/S	N/S
FAS pathway	23	N/S	N/S	N/S	N/S	N/S	N/S	N/S	N/S	N/S	N/S	N/S	N/S
ST FAS signaling pathway	56	N/S	N/S	N/S	N/S	N/S	N/S	.01*	.17*	N/S	N/S	N/S	N/S
Caspase pathway	20	N/S	N/S	N/S	N/S	N/S	N/S	0*	.10*	N/S	N/S	N/S	N/S
SA caspase cascade	15	N/S	N/S	N/S	N/S	N/S	N/S	N/S	N/S	N/S	N/S	N/S	N/S
PKC pathway	6	N/S	N/S	N/S	N/S	N/S	N/S	.01*	.14*	N/S	N/S	N/S	N/S
Passerini oxidation	16	N/S	N/S	N/S	N/S	N/S	N/S	0*	.11*	N/S	N/S	N/S	N/S
JNK up	25	N/S	N/S	N/S	N/S	N/S	N/S	0*	.07*	N/S	N/S	N/S	N/S
Ceramide pathway	20	N/S	N/S	N/S	N/S	N/S	N/S	N/S	N/S	N/S	N/S	N/S	N/S
BAD pathway	18	N/S	N/S	N/S	N/S	N/S	N/S	N/S	N/S	N/S	N/S	N/S	N/S
P53 pathway	15	N/S	N/S	N/S	N/S	N/S	N/S	N/S	N/S	N/S	N/S	N/S	N/S
P53 hypoxia pathway	15	N/S	N/S	N/S	N/S	N/S	N/S	N/S	N/S	N/S	N/S	N/S	N/S

Candidate mRNA levels of selected apoptotic genes (*TNF-α*, *TRAIL*, and *caspase-4*) were validated by real-time qRT-PCR (Table 2). The 16-day time point was chosen for this comparison because it is the time point where most changes were observed. Microarray assessment of *TNF-α*, *TRAIL*, and *caspase-4* expression had shown that the mRNA levels of these genes were enhanced in diabetic animals by 1.7- to 2.0-fold (*p* < .05). When examined by real-time PCR the results indicated a 1.8- to 3.8-fold elevation in the diabetic group, thus confirming increased mRNA levels (*p* < .05).

**Table 2:** mRNA Levels Obtained by Microarray or Real-Time PCR

	Real-time PCR (relative intensity)			Microarray (relative intensity)		
		
	Normal	Diabetic	Fold change	Normal	Diabetic	Fold change
*TNF-α*	2.5 ± 0.1	9.5 ± 1.1	3.8	2.3 ± 0.3	4.6 ± 0.6	2.0
*TRAIL*	1.2 ± 0.1	3.0 ± 0.6	2.5	3.8 ± 0.8	6.5 ± 1.4	1.7
*Caspase-4*	1.1 ± 0.1	2.0 ± 0.1	1.8	1.8 ± 0.1	3.4 ± 0.4	1.9

*Note*: Microarray analysis or real-time PCR was carried out three times, as described earlier, on day 16 fracture-healing specimens. The fold change represents the values of the diabetic compared with normoglycemic specimens. Each value represents the mean ± SEM of triplicate samples. In each case, the diabetic value was significantly higher than the normoglycemic value (*p* < .05).

**Fig. 1:**
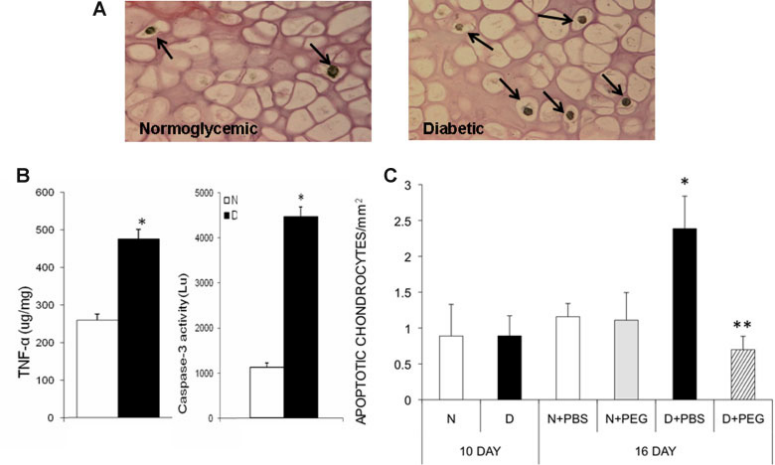
TNF-α protein levels and caspase-3 activity are increased by diabetes in healing fractures, and TNF inhibition reverses diabetes-enhanced chondrocyte apoptosis. (*A*) Histologic images of cartilage from normoglycemic and diabetic mice (original magnification ×400). Arrows indicate TUNEL^+^ chondrocytes. (*B*) Protein was extracted from fracture calluses and tested for TNF-α by ELISA (*right panel*) and for caspase-3 activity by a luminescent assay (*left panel*). Each value represents the mean ± SEM of triplicate samples, and each assay was carried out three times with similar results. *A significant difference between normoglycemic and diabetic groups (*p* < .05). Sections from fracture calluses were examined by the TUNEL assay and counterstained with safranin-O/fast green to identify cartilage. (*C*) Quantitative analysis of TUNEL^+^ cells in diabetic mice (*D*), matched normoglycemic control mice (*N*). Normoglycemic or diabetic mice were treated with pegsunercept (PEG) or vehicle alone (PBS). Data are expressed as mean ± SEM. *A significant difference from the diabetic group (*p* < .05). **Significant difference between diabetic and pegsunercept-treated diabetic mice (*p* < .05).

Comparison studies of callus and cartilage area demonstrated that diabetes resulted in a decrease in callus and cartilage area, and TNF-α inhibition resulted in reversal of the effect of diabetes (*p* < .05; Supplemental Table S1). Subsequent studies were carried out to functionally assess chondrocyte apoptosis, TNF-α at the protein level, and caspase-3 at the level of activity on day 16. Increased TUNEL^+^ chondrocytes, which were predominantly hypertrophic chondrocytes, were consistently observed at higher levels in the diabetic group (Fig. 1*A*). Higher levels of apoptosis coincided with a 1.8-fold higher level of TNF-α and a 3.9-fold higher level of caspase-3 activity in the fracture calluses in the diabetic group compared with the normoglycemic group (*p* < .05; Fig. 1*B*). The increase in chondrocyte apoptosis was established by quantitative analysis. Diabetes enhanced chondrocyte apoptosis on day 16 (*p* < .05) but did not on day 10 (Fig. 1*C*). Given that diabetic mice had elevated TNF-α protein and mRNA levels and that gene sets related to TNF signaling were enhanced by diabetes (Table 1), a potential mechanism for the increased levels of apoptosis that were seen in diabetes was investigated by treating mice with the TNF-specific inhibitor pegsunercept. When diabetic mice were treated with pegsunercept, there was a 76% decrease in chondrocyte apoptosis in the diabetic group so that the levels returned to those seen in normal animals (*p* < .05; Fig. 1*C*). In contrast, there was no significant difference in chondrocyte apoptosis in normoglycemic mice treated with pegsunercept compared with vehicle-treated controls (*p* > .05). On day 22, the amount of cartilage was minimal, precluding the accurate counting of apoptotic chondrocytes.

**Fig. 2:**
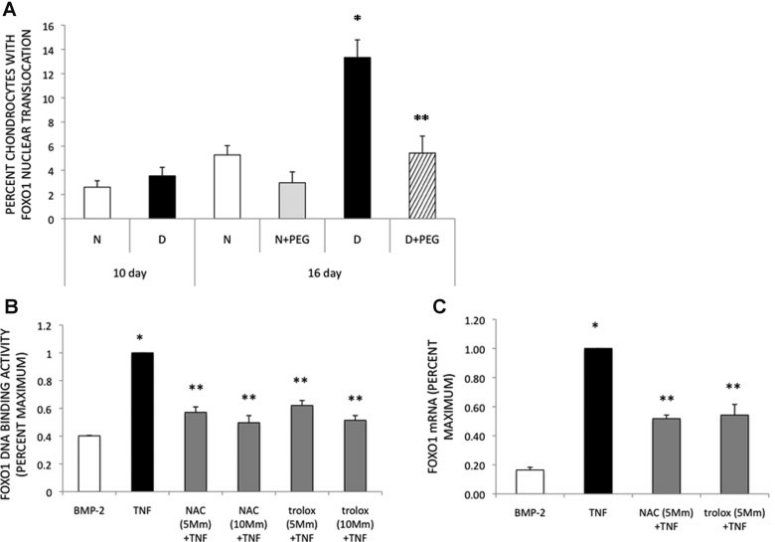
Diabetes increases FOXO1 nuclear localization in chondrocytes of healing fractures, and inhibition of ROS reduces TNF-α-stimulated FOXO1 DNA binding and *FOXO1* mRNA. (*A*) Transverse sections from calluses were incubated with anti-FOXO1 IgG or matched-control IgG, and nuclear localization was assessed by confocal scanning laser microscopy by counting the percentage of chondrocytes with unambiguous FOXO1 nuclear localization. ATDC5 cells were stimulated with BMP-2 and TNF-α. This was followed by treatment of cells by the ROS inhibitors NAC and Trolox. (*B*) FOXO1 DNA-binding activity was measured using ELISA. (*C*) *FOXO1* mRNA was isolated and measured using qPCR. Data are expressed as mean ± SEM. *A significant difference from the diabetic group and a significant increase with TNF-α stimulation (*p* <.05). **Significant difference between diabetic and pegsunercept-treated diabetic mice and significant reduction with ROS inhibitors (*p* < .05).

To determine whether FOXO1 was affected by diabetes, we examined FOXO1 nuclear localization that occurred in chondrocytes in diabetic fractures by confocal laser scanning microscopy (Supplemental Fig. S1). When FOXO1 is activated, it translocates to the nucleus, and on deactivation, it is quickly transported out of the nucleus. There were no differences in FOXO1 nuclear localization between diabetic mice and normoglycemic mice on day 10 (*p* > .05). On day 16, diabetes caused a 2.5-fold increase in the percentage of chondrocytes with FOXO1 nuclear localization compared with matched normoglycemic controls (*p* < .05; Fig. 2*A*). Inhibition of TNF in the diabetic group resulted in a 59.3% decrease in the percentage of chondrocytes that had FOXO1 nuclear localization (*p* < .05). In contrast, TNF inhibition had no significant effect on FOXO1 nuclear localization in normoglycemic mice (*p* > .05).

To better understand the mechanistic process of chondrocyte apoptosis, TNF-α−stimulated chondrocyte apoptosis was investigated in vitro by examining the role of FOXO1, which has been shown to mediate proapoptotic gene expression.(20,21) RNAi studies were performed in BMP-stimulated ATDC5 cells that have a chondrogenic phenotype.(36) TNF-α increased *FOXO1* mRNA levels 3.8-fold and DNA-binding activity almost 2-fold (*p* < .05; Table 3). When transfected with siRNA and then stimulated with TNF-α, *FOXO1* mRNA levels were reduced by 73% compared with scrambled siRNA (*p* < .05). TNF-α-stimulated *FOXO1* DNA-binding activity was reduced 63% by *FOXO1* siRNA compared with scrambled siRNA (*p* < .05). Scrambled siRNA had no effect on *FOXO1* mRNA or DNA-binding activity compared with cells that were not transfected (Table 3).

**Table 3:** *FOXO1* mRNA Levels and DNA-Binding Activity After Silencing With *FOXO1* siRNA

	*FOXO1* mRNA	FOXO1 DNA-binding activity
Negative control	1.00 ± 0.00	1.00 ± 0.0
BMP	0.93 ± 0.08	1.0 ± 0.1
BMP + TNF	3.7 ± 0.3*	1.9 ± 0.2*
BMP + TNF + SCR siRNA	3.4 ± 0.1*	1.8 ± 0.2*
BMP + TNF + FOXO1 siRNA	1.0 ± 0.3**	0.7 ± 0.1**

*Note*: ATDC5 cells were stimulated with BMP-2 and with TNF-α. This was followed by transfection with either *FOXO1* siRNA or scrambled siRNA. mRNA then was isolated, and the level *FOXO1* mRNA was measured. FOXO1 DNA-binding activity also was measured using ELISA. Data expressed as mean ± SEM.

* Significant increase with TNF-α stimulation (*p* < .05).

** Significant reduction with *FOXO1* siRNA (*p* < .05).

Since FOXO1 has been linked to oxidative stress,(17) we determined whether inhibition of reactive oxygen species (ROS) affected the capacity of TNF-α to induce FOXO1. When ROS was inhibited by NAC or by Trolox, the level of *FOXO1* DNA-binding activity was reduced by 60%, which represents a 90% decrease in the amount stimulated by TNF-α (Fig. 2*A* and 2*B*). *FOXO1* mRNA levels were reduced by 50% by either of the ROS inhibitors, which represents an 80% decrease in the mRNA levels stimulated by TNF-α (Fig. 2*C*).

**Fig. 3:**
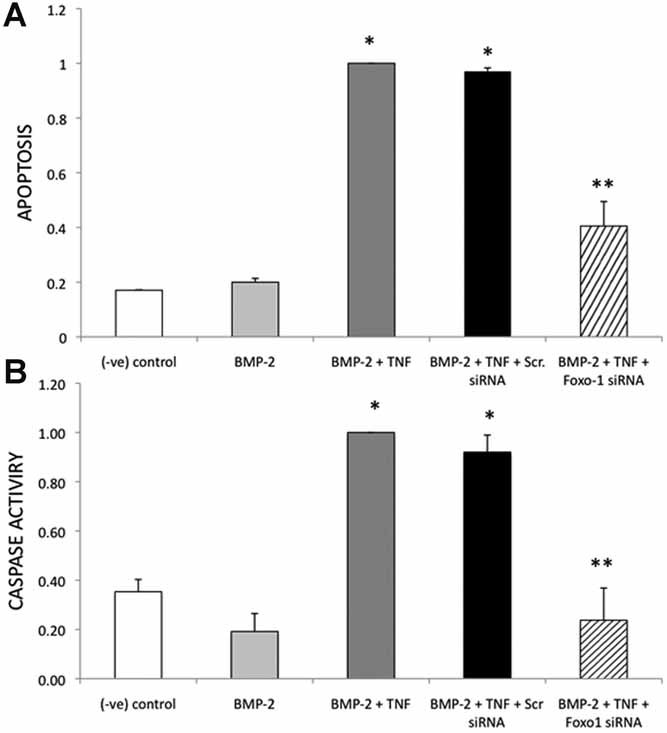
TNF-induced apoptosis and caspase-3/7 activity in ATDC5 chondrogenic cells depends on FOXO1. BMP2-stimulated ATDC5 cells were transfected with either *FOXO1* siRNA or scrambled siRNA and stimulated with TNF-α (20 ng/mL). (*A*) Apoptosis was measured by histone-associated cytoplasmic DNA and (*B*) caspase-3/7 activity with a luminescent substrate. The data presented are the mean of three independent experiments ± SEM and are shown as percent of maximum stimulation. *Significant increase with TNF-α σtimulation (*p* < .05). **Significant reduction with *FOXO1* siRNA (*p* < .05).

The role of FOXO1 on TNF-α-induced apoptosis and caspase activity was assessed by siRNA. TNF-α stimulated a 5-fold increase in apoptosis, whereas FOXO1 knockdown decreased that level of apoptosis by almost 60% (*p* < .05; Fig. 3*A*). TNF-α also increased caspase-3/7 activity by 5-fold in ATDC5 cells compared with cells not incubated with TNF-α (*p* < .05). FOXO1 knockdown reduced caspase-3/7 activity by 75% when compared with cells transfected with scrambled siRNA (*p* < .05; Fig. 3*B*). There was no significant difference between scrambled siRNA transfected and nontransfected cells (*p* > .05).

**Fig. 4:**
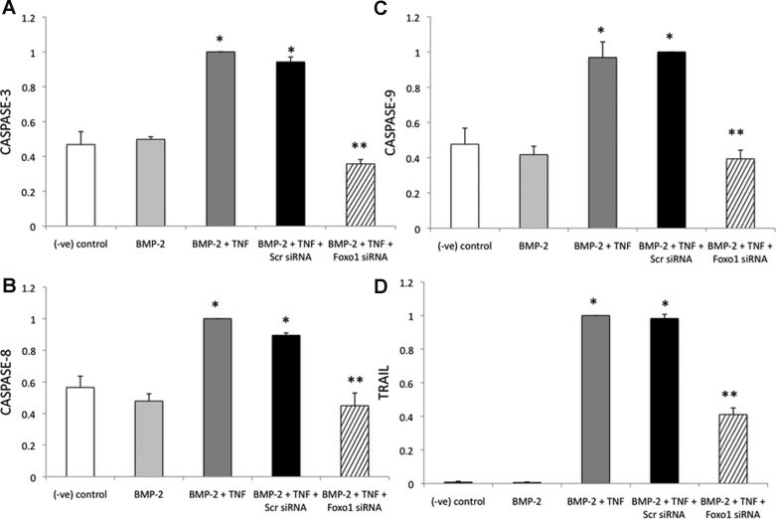
*FOXO1* siRNA decreases TNF-α-induced apoptosis in BMP-2-stimulated cells at mRNA levels. ATDC5 cells were treated with BMP-2 and then stimulated with TNF-α. Cells treated with TNF-α were pretransfected with either *FOXO1*-specific or scrambled siRNA. Total mRNA levels of selected proapoptotic genes were tested by real-time PCR. (*A*) *Caspase-3*. (*B*) *Caspase-8*. (*C*) *Caspase-9*. (*D*) *TRAIL*. The data presented are the mean of three independent experiments ± SEM and are shown as percent of maximum stimulation. *Significant increase with TNF-α stimulation (*p* < .05). **Significant reduction with *FOXO1* siRNA (*p* < .05).

To establish whether FOXO1 mediated TNF stimulation of proapoptotic genes, real-time PCR was carried out. TNF-α stimulated a 2-fold increase in *caspase-3* mRNA in ATDC5, and FOXO1 knockdown decreased *caspase-3* mRNA levels by over 60% compared with scrambled siRNA (*p* < .05; Fig. 4*A*). *Caspase-8* mRNA was increased 1.9-fold in ATDC5 cells with TNF-α stimulation, which was reduced by 49% when FOXO1 was knocked down compared with scrambled siRNA (*p* < .05; Fig. 4*B*). Similar results were obtained with *caspase-9*, which was increased 2.3-fold in cells with TNF-α stimulation (*p* < .05). FOXO1 knockdown reduced this by 62% (*p* < .05; Fig. 4*C*). *TRAIL* was upregulated 200-fold in TNF-α-stimulated ATDC5 cells compared with cells without TNF-α (*p* < .05). *FOXO1* siRNA reduced this by 59% compared with scrambled siRNA (*p* < .05; Fig. 4*D*). These results suggest that FOXO1 mediates mRNA levels of proapoptotic genes induced by TNF-α in cells with a hypertrophic chondrocyte phenotype.

**Fig. 5:**
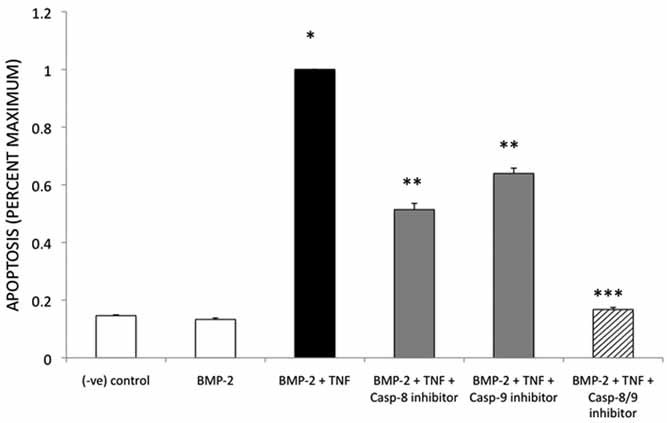
Inhibition of apoptosis by inhibition of caspase-8 and caspase-9 in ATCD5 cells. ATDC5 cells were treated with BMP-2 and then stimulated with TNF-α. Cells then were treated with caspase-8 inhibitor or caspase-9 inhibitor or both. Apoptosis was measured by histone-associated cytoplasmic DNA. The data presented are the mean of three independent experiments ± SEM and are shown as percent of maximum stimulation. *Significant increase with TNF-α stimulation (*p* < .05). **Significant reduction compared with TNF-α stimulation (*p* < .05). ***Significant reduction compared with treatment with one inhibitor (*p* <.05).

To establish whether *caspase-8* and *caspase-9*, whose mRNA levels were regulated by FOXO1, played a prominent role in TNF-α-induced chondrocyte apoptosis, specific peptide inhibitors were used. Inhibition of *caspase-8* reduced TNF-α-stimulated apoptosis by approximately 40%, and inhibition of *caspase-9* reduced TNF-α-stimulated apoptosis by 50%, both of which were significant (*p* < .05). However, when both inhibitors were used together, apoptosis was reduced by 85% (*p* < .05), indicating that both caspases participate in the apoptotic process (Fig. 5). This suggests that the extracellular and intracellular apoptotic pathways are involved in mediating TNF-α-induced cell death in chondrogenic cells.

**Fig. 6:**
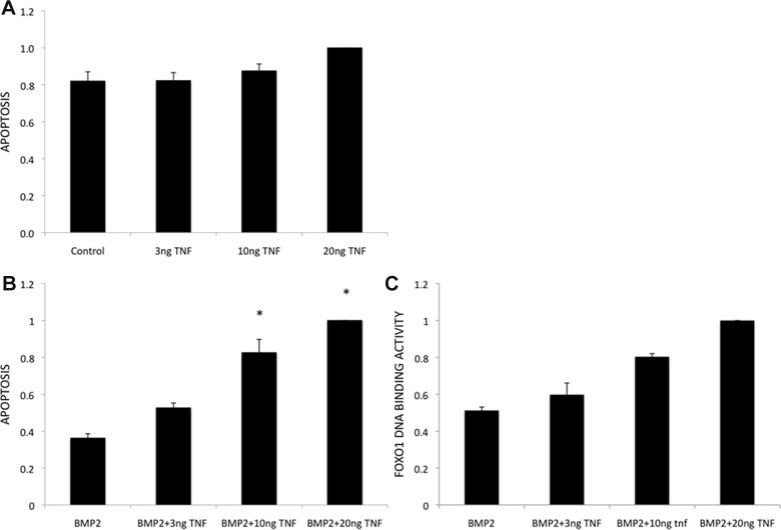
TNF-α stimulates apoptosis in a dose-response manner in C3H10T1/2 cells only after they acquire a chondrogenic phonotype. Cells were grown with or without BMP-2 treatment and stimulated with TNF-α in a dose-response manner, and apoptosis and FOXO1 DNA binding were measured. (*A*) Apoptosis assay in undifferentiated C3H10T1/2 cells. (*B*) Apoptosis assay in differentiated C3H10T1/2 cells. (*C*) FOXO1 DNA-binding activity in differentiated C3H10T1/2 cells. The data presented are the mean of three independent experiments ± SEM and are shown as percent of maximum stimulation. *Significant increase with TNF-α stimulation (*p* < .05).

**Fig. 7:**
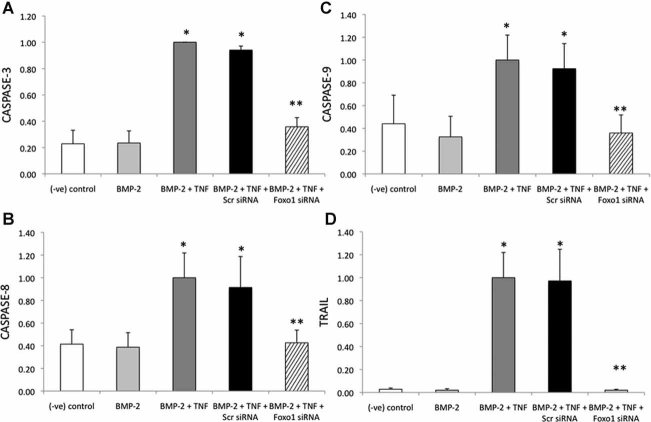
*FOXO1* siRNA decreases TNF-α-induced apoptosis in BMP-2-stimulated cells at mRNA levels. C3H10T1/2 cells were treated with BMP-2 and then stimulated with TNF-α. Cells treated with TNF-α were pretransfected with either *FOXO1-*specific or scrambled siRNA. Total mRNA levels of selected proapoptotic genes were tested by real-time PCR. (*A*) *Caspase-3*. (*B*) *Caspase-8*. (*C*) *Caspase-9*. (*D*) *TRAIL*. The data presented are the mean of three independent experiments ± SEM and are shown as percent of maximum stimulation. *Significant increase with TNF-α stimulation (*p* < .05). **Significant reduction with *FOXO1* siRNA (*p* < .05).

Selected results obtained with ATDC5 cells were confirmed with C3H10T1/2 cells. C3H10T1/2 cells were incubated with BMP-2 and differentiated to chondrogenic cells, as shown by several-fold induction of mRNA for *collagen II* and *collagen X* (data not shown). In the absence of BMP-2 stimulation, TNF-α did not stimulate apoptosis (Fig. 6*A*). However, when these cells acquired a chondrogenic phenotype, TNF-α stimulated a dose-dependent increase in apoptosis in both (Fig. 6*B*). When tested for induction of proapoptotic genes, TNF-α stimulated a 3- to 5-fold increase in *caspase-3*, *caspase-8*, and *caspase-9* mRNA levels and more than a 50-fold increase in *TRAIL* mRNA levels in C3H10T1/2 cells with a chondrogenic phenotype (Fig. 7). In each case, *FOXO1* siRNA knocked down the mRNA level by more than 60%, which was significant for each gene (*p* < .05). In control experiments, *FOXO1* siRNA significantly knocked down TNF-α-stimulated *FOXO1* mRNA levels by more than 80%, whereas scrambled siRNA had no effect (data not shown).

## Discussion

Relatively little is known about the impact of diabetes on the transition from cartilage to bone during fracture healing. Our group has previously reported that diabetic fracture healing is associated with smaller calluses, premature loss of cartilage, and increased osteoclastic activity.(13,27) Recently, we also have shown that inhibiting TNF-α reverses many of these effects.(22) We focused on a potentially important event, chondrocyte apoptosis. To investigate pathways that may affect apoptosis in diabetic animals, large-scale transcriptional profiling analysis was carried out on total RNA that was isolated from normal and diabetic callus tissue at various time points, and the nature of expressed genes was assessed by comparisons of gene expression sets that were preferentially observed in the two sets of callus tissues. These results showed that diabetes caused the upregulation of several apoptotic pathways, particularly during the transition from cartilage to bone on day 16. The upregulation of apoptotic pathways agreed well with the increase in caspase-3 activity noted in the diabetic group and the enhanced number of apoptotic chondrocytes. At an earlier time point, there was no upregulation of apoptotic gene sets or chondrocyte apoptosis in the diabetic group. This may be significant because enhanced or premature chondrocyte cell death may trigger subsequent events that accelerate degradation of cartilage.(17) It was shown in articular chondrocytes that TNF-α-induced apoptosis was associated with increased expression of genes related to matrix degradation such as *MMP* and macrophage colony stimulating factor (*MCSF*).(38) Similarly, in the absence of TNF-α during bone repair, expression of these genes is downregulated.(39) This link is supported by findings that mRNA levels of *MCSF* and other proresorptive cytokines are also elevated on day 16 in diabetic compared with normal animals.(13)

High levels of TNF have been linked to a number of osseous disorders, including osteoporosis, osteolysis, osteoarthritis, and rheumatoid arthritis.(40–42) At both the mRNA and protein levels, diabetes caused a significant increase in TNF-α during fracture healing. Chondrocytes are particularly sensitive to TNF-induced apoptosis,(38,43) consistent with results that TNF alone stimulated apoptosis in C3H10T1/2 cells with a BMP-2-induced chondrogenic phenotype but not in undifferentiated C3H10T1/2 mesenchymal cells. When diabetic mice were treated with a TNF-specific inhibitor, we noted a significant reduction in chondrocyte apoptosis in diabetic mice but not in normoglycemic mice. This would indicate that the increase in TNF-α is particularly important in the diabetic animals and drives apoptosis of chondrocytes. In parallel studies, TNF-α was produced at the highest levels in hypertrophic chondrocytes in diabetic calluses during fracture repair (data not shown). This would suggest an autocrine mechanism for TNF-stimulated apoptosis of hypertrophic chondrocytes. We have previously reported that when TNF receptor signaling is completely abrogated by genetic deletion, chondrocyte apoptosis is delayed, and we have postulated that this delay interferes with fracture healing.(44) In vivo and in vitro experiments performed here indicate that TNF plays a role in the expression of proapoptotic genes enhanced by diabetes and that TNF-stimulated apoptosis in chondrocytes is mediated by FOXO1. It is possible that this occurs through an autocrine mechanism because we previously reported that chondrocytes in diabetic fracture healing exhibit increased expression of TNF-α in vivo,(22) and others have shown that chondrocytes undergoing apoptosis in vivo express caspase 3 and 9, both of which are targets of FOXO1.(19,45,46) These results indicate the TNF initiates the early phase of chondrocyte apoptosis, but at the later phases, other factors such as FasL are more likely to have a significant role in chondrocyte apoptosis in normoglycemic animals.

It has been shown in vivo that diabetes increases FOXO1 DNA-binding activity and increases FOXO1 nuclear translocation in chondrocytes in fracture repair.(22) This is also shown in this study, but when TNF was inhibited, FOXO1 nuclear translocation was reduced, which may indicate that the effects of TNF on apoptosis is mediated through FOXO1. To investigate further the mechanism by which TNF could induce chondrocyte apoptosis, in vitro studies were carried out. Two cell lines with a BMP-2-induced chondrogenic phenotype were examined and produced similar results. TNF-α stimulated apoptosis and proapoptotic gene expression in both chondrogenic cells that was significantly reduced by FOXO1 knockdown. Interestingly, TNF did not induce apoptosis in undifferentiated C3H10T1/2 cells (Fig. 6) and did not enhance mRNA levels in the undifferentiated cells (unpublished data). Thus the acquisition of a chondrogenic phenotype renders cells sensitive to TNF-α-induced apoptosis and FOXO1 activation, consistent with knockdown experiments in which FOXO1 played an important role in mediating TNF-induced apoptosis and proapoptotic gene expression. FOXO1 induces the expression of a large number of proapoptotic extracellular and intracellular mediators such as FasL, TRAIL, and caspases.(19,47) Because of the increased expression of proapoptotic factors, caspase activity is also increased, and apoptosis is induced.(19,47) In the present study, silencing FOXO1 in chondrogenic cells not only inhibited apoptosis but also inhibited *TRAIL*, *caspase-3*, *caspase-8*, and *caspase-9*.

Diabetes-enhanced apoptosis is associated with diabetic complications. Diabetes increases the rate of fibroblast apoptosis stimulated by a bacteria-induced wound, and the higher level of apoptosis contributes to an impaired wound-healing response.(48) There is also evidence of increased apoptosis of pericytes and endothelial cells in retinas of diabetic animals, which was found to be a contributing factor to diabetic retinopathy and caused in part by increased levels of TNF.(49–52) Thus TNF dysregulation caused by diabetes may affect a number of different tissues, including cartilage, during fracture healing. This may lead to greater chondrocyte apoptosis, which potentially could affect the cartilage in the diabetic animals. It is striking that diabetic fracture healing is also characterized by increased production of proresorptive factors and accelerated formation of osteoclasts and cartilage degradation.(13,27)
