# Supplementary figures and images for: TNF-α Mediates Diabetes-Enhanced Chondrocyte Apoptosis During Fracture Healing and Stimulates Chondrocyte Apoptosis Through FOXO1
Source: J Bone Miner Res. 2010 Feb 8;25(7):1604–15. doi: 10.1002/jbmr.59 (PMC3154002; doi:10.1002/jbmr.59)

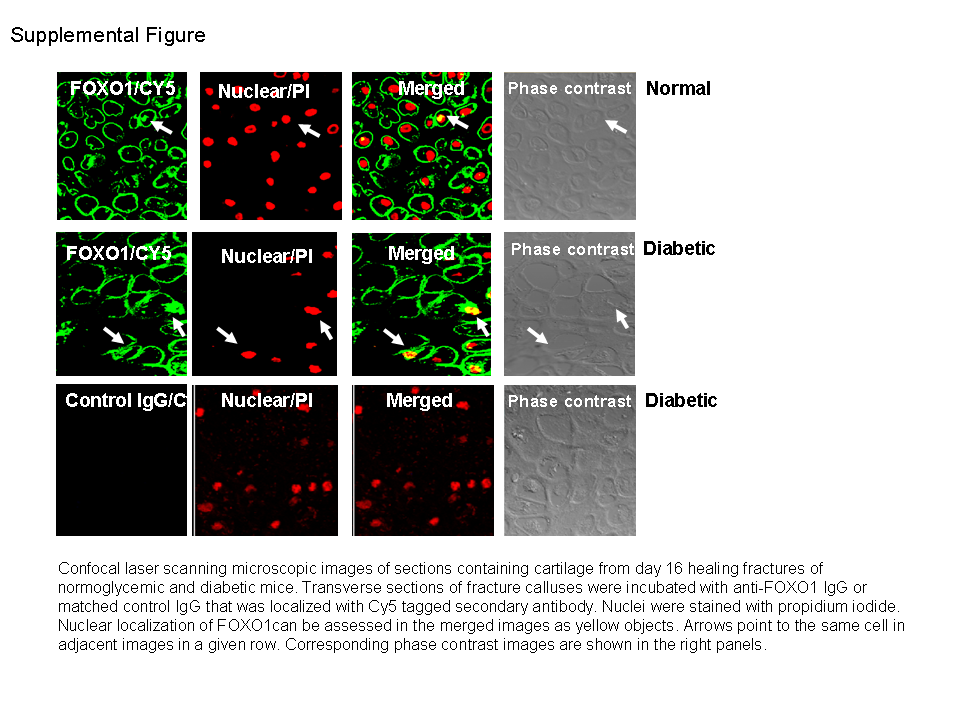

Supplement: Supplementary file 1 [file jbmr0025-1604-SD1.tif]
