# Supplementary material for: TNF-α Mediates Diabetes-Enhanced Chondrocyte Apoptosis During Fracture Healing and Stimulates Chondrocyte Apoptosis Through FOXO1
Source: J Bone Miner Res. 2010 Feb 8;25(7):1604–15. doi: 10.1002/jbmr.59 (PMC3154002; doi:10.1002/jbmr.59)
Supplement: Supplementary file 2 [file jbmr0025-1604-SD2.doc]

**Supplemental Table**: Changes in callus and cartilage area in response diabetes and treatment with insulin and TNF inhibitor.

|  | **Diabetic/Normal (Fold)** | **TNF inhibitor/Diabetic (Fold)** |
| --- | --- | --- |
| **Callus Area (mm^2^)** | 0.75 * | 1.2 * |
| **Cartilage Area (mm^2^)** | 0.54 * | 1.5 * |

Comparison of callus and cartilage size in diabetic, normoglycemic and diabetic insulin treated mice with femoral fracture on day 16. Callus area was measured in H&E stained cross-sections obtained from 3 points sampled at the fracture line and 0.5mm proximal and distal and presented as the sum of these three sites. The area of cartilage within each callus was measured in safranin-O/fast green stained sections in the same manner. * indicates a significant difference between normal and diabetic (P<0.05).
